# Communications

**Published:** 1903-01

**Authors:** E. B. Ackerman

**Affiliations:** Chairman; Brooklyn


### COMMUNICATION.

BROOKLYN, January 4, 1903.

DR. W. HORACE HOSKINS, EDITOR VETERINARY JOURNAL,
3452 Ludlow Street, Philadelphia, Pa.

DEAR DOCTOR: The Committee on Intelligence and Education is desirous of obtaining the following information and thinks it might obtain the quickest results through your valuable magazine, and therefore address this communication to you, so that you might publish this in your next issue. We want to know the name of each and every veterinary college in the United States and Canada, whether it is an independent school or whether it is connected with some university or agricultural college; also the name of the dean or secretary, and the correct address of the school and its officers, so that this committee can compile a correct list of schools and get in direct communication with each of them.

We also want to obtain a correct list of all boards of examiners connected with the regents in each State with the post-office address of such board and its president or secretary, so that we can communicate directly with them also.

The committee would also like to find out if there is any particular class or kind of literature that any State or locality would like this committee to bring prominently before any public officials or before the public through the aid of the public press, or to the attention of any health or agricultural departments, or any cattle or live-stock boards; this committee will do its best to get such literature in shape to present to the proper people in the best possible manner. Kindly state the character of the literature and for what purpose, with the name of the person, body, or paper that you desire us to get to, with the address of each and all that you desire us to reach.

To get this information accurately it will be necessary that this committee hear from each State and Canada, so that it will be complete, correct, and accurate, and with proper addresses.

The committee respectfully calls this to the attention of the resident State secretaries and asks them to co-operate with this committee and to make it their business to furnish this information accurately and as soon as possible, so that we may go ahead on the lines suggested, and act according to the resolutions passed at the last meeting of our association.

Trusting you will have space to insert this in the next issue of your valuable magazine, I remain,

Very truly yours,

E. B. Ackerman,
Chairman.

